# Stathmin inhibits proliferation and differentiation of dental pulp stem cells via sonic hedgehog/Gli

**DOI:** 10.1111/jcmm.13621

**Published:** 2018-04-14

**Authors:** Dandan Ma, Haiyue Yu, Shuaimei Xu, He Wang, Xiaoyi Zhang, Tingting Ning, Buling Wu

**Affiliations:** ^1^ Department of Stomatology Nanfang Hospital Southern Medical University Guangzhou China; ^2^ College of Stomatology Southern Medical University Guangzhou China; ^3^ Department of Operative and Endodontic Dentistry Stomatological Hospital Southern Medical University Guangzhou China

**Keywords:** cell differentiation, cell proliferation, odontoblastic mineralization, osteogenic mineralization, sonic hedgehog, Stathmin

## Abstract

The mineralization of dental pulp stem cells is an important factor in the tissue engineering of teeth, but the mechanism is not yet obvious. This study aimed to identify the effect of Stathmin on the proliferation and osteogenic/odontoblastic differentiation of human dental pulp stem cells (hDPSCs) and to explore whether the Shh signalling pathway was involved in this regulation. First, Stathmin was expressed in the cytoplasm and on the cell membranes of hDPSCs by cell immunofluorescence. Then, by constructing a lentiviral vector, the expression of Stathmin in hDPSCs was inhibited. Treatment with Stathmin shRNA (shRNA‐Stathmin group) inhibited the ability of hDPSCs to proliferate, as demonstrated by a CCK8 assay and flow cytometry analysis, and suppressed the osteogenic/odontoblastic differentiation ability, as demonstrated by alizarin red S staining and osteogenic/odontoblastic differentiation‐related gene (ALP, BSP, OCN, DSPP) activity, compared to that of hDPSCs from the control shRNA group. Molecular analyses showed that the Shh/GLI1 signalling pathway was inhibited when Stathmin was silenced, and purmorphamine, the Shh signalling pathway activator, was added to hDPSCs in the shRNA‐Stathmin group, real‐time PCR and Western blotting confirmed that expression of Shh and its downstream signalling molecules PTCH1, SMO and GLI1 increased significantly. After activating the Shh signalling pathway, the proliferation of hDPSCs increased markedly, as demonstrated by a CCK8 assay and flow cytometry analysis; osteogenic/odontoblastic differentiation‐related gene (ALP, BSP, OCN, DSPP) expression also increased significantly. Collectively, these findings firstly revealed that Stathmin‐Shh/GLI1 signalling pathway plays a positive role in hDPSC proliferation and osteogenic/odontoblastic differentiation.

## 1 INTRODUCTION

Dental pulp stem cells (DPSCs) derived from dental pulp are capable of differentiating into odontoblasts, chondroblasts, osteoblasts, angiogenic and active neurons under the appropriate environmental conditions.^1^, ^2^, ^3^, ^4^, ^5^, ^6^ Adult human DPSCs are considered as a valuable source for pulpo‐dentinal complex regeneration in tooth loss^7^ and also a new tool in bone tissue engineering which can fabricate vascularized woven bone tissue.^8^ Although the mineralization‐related molecules involved in DPSC differentiation into odontoblasts/osteoblasts (such as BMP, SHH, TRPM7, etc.) are known,^9^, ^10^, ^11^ knowledge of their mechanism remains limited. Therefore, important issue for efficient tooth regeneration is to target hDPSCs to promote their osteogenic/odontoblastic differentiation potential.

Stathmin (also called OP18), a key endogenous regulator of microtubule dynamic,^12^, ^13^, ^14^ is a ubiquitously expressed, small cytosolic phosphoprotein that was originally identified as an important factor involved in regulating cell proliferation.^15^, ^16^ Microtubules, which are involved in bone metabolism and microtubule assembly modulation, are major components of the cytoskeleton and can regulate changes in bone mass. Microtubule assembly affects bone mass by preventing the proteolytic degradation of GLI2, a major mediator of hedgehog signalling, and in turn stimulates bone morphogenetic protein 2 expression in osteoblast cells.^17^ Stathmin plays an essential role in the maintenance of postnatal bone mass by regulating both osteoblast and osteoclast functions in bone. Although Stathmin seems to be a crucial regulator responsible for the initiation and mineralization of bone, its role in dental pulp is poorly documented.

In our previous study, we showed higher expression of Stathmin in DPSCs than in DPSCs derived from caries^18^, ^19^ using 2‐D DIGE and Western blotting. Based on similar intrinsic properties between the mineralization of DPSCs and Bone Mesenchymal Stem Cells (BMSCs), the regulation of osteoblast mineralization by microtubules via the Shh signalling pathway could have multiple functions in tooth development and could enhance the proliferation and odontoblastic differentiation of human dental pulp cells via the Shh/GLI1 signalling pathway.^20^, ^21^, ^22^ We have been suggested that Stathmin, as an important microtubule regulator, plays an important role in hDPSC osteogenic/odontoblastic differentiation via the Shh signalling pathway.

## 2 MATERIALS AND METHODS

### 2.1 Human dental pulp stem cells culture

Human dental pulp tissues were collected from clinically extracted, periodontally healthy and noncarious human mandibular third molars from Nanfang Hospital affiliated Southern Medical University, Guangzhou, China. Human subjects who participated in the study all provided written informed consent, which was approved by the Ethics Committee of Nanfang Hospital. All patients were performed under local anaesthesia by nerve‐block of the inferior alveolar nerve, lingual nerve and buccal nerve with two 1.8 mL cartridges of 4% articaine with epinephrine 1:200 000.^23^ Before the tooth extracted, the dental crown is handled with 0.3% chlorexidin gel (Forhans, NY, USA) for 2 minutes. Dental pulp was gently removed from the crown and root and dissected into 1 mm^3^ pieces with ophthalmic scissors. Fragments were digested in 3 mg/mL collagenase type I (Sigma, St Louis, USA) for 1 hour at 37°C. The solution was then filtered with 70 μm Falcon strainers (Becton & Dickinson, Buccinasco, Milan, Italy) to remove the tissue pieces not digested. Then, cells were rinsed in Dulbecco's modified Eagle's medium (DMEM) supplemented with 10% foetal bovine serum, 100 U/mL penicillin and 100 μg/mL streptomycin (HyClone, NY, USA). After centrifugation, the supernatant was removed, and the tissue blocks were added to DMEM low glucose medium containing 10% foetal bovine serum and then transferred to 25 cm^2^ culture flasks (Corning Inc., NY, USA), which were inverted and incubated in a 5% CO_2_ atmosphere at 37°C. The culture flasks were turned over 24 hours later. The individual hDPSCs were released from the pulp after 3‐5 days.^24^ DPSCs were enriched by collecting multiple colonies. DPSCs at passage 3 were used in each experiment.

### 2.2 Immunocytochemistry

Human dental pulp stem cells cultured in a confocal dish were fixed in 4% paraformaldehyde for 40 minutes and washed three times with PBS. Then, 0.25% (v/v) Triton X‐100 in PBS was used to permeabilize the cell membranes for 15 minutes at room temperature. The cells were rinsed in 2% BSA for 30 minutes and then incubated overnight with rabbit anti‐human Stathmin monoclonal antibody (Abcam, Cambridge, UK) at a dilution of 1:100 in 2% BSA in PBS at 4°C.

After they were washed in PBS, the hDPSCs were covered with FITC‐labelled goat anti‐rabbit IgG antibody (1:100) and DAPI. Immunofluorescence was detected using an Olympus FV 10i‐W confocal microscope (Olympus, Tokyo, Japan).

### 2.3 Lentiviral vector cell transduction

A lentivirus containing silencing Stathmin shRNA and the green fluorescent protein (GFP) gene and a negative control consisting of a scrambled sequence and GFP were constructed by the GenePharma Company (GenePharma, Shanghai, China). hDPSCs were infected with shRNA‐Stathmin and shRNA‐Ctrl lentiviruses at a multiplicity of infection of 50.

### 2.4 Purmorphamine treatment analysis

After transfection with shRNA‐Stathmin lentiviruses, hDPSCs were treated with the Shh signalling pathway activator purmorphamine (Selleck, Houston, USA) at a final concentration of 1 μmol/L for 4 days. shRNA‐Stathmin transfected hDPSCs without purmorphamine treatment were regarded as the control group (n = 3 for each group).

### 2.5 CCK8 assay

hDPCSs transduced with shRNA‐Stathmin lentiviruses, shRNA‐Stathmin lentiviruses + purmorphamine and shRNA‐Ctrl lentiviruses were seeded at 2 × 10^3^ cells in 96‐well culture plates for the CCK8 assay. Detection was performed on days 1, 3, 5 and 7. Then, 10 μL of CCK8 reagent was added to each culture well and the cells were incubated avoiding light for 2 hours at 37°C. The absorbance was measured using a SpectraMax M5 multi—functional microplate reader (BD Falcon, San Jose, USA) at a wavelength of 490 nm. The CCK8 assay was repeated in triplicate.

### 2.6 Flow cytometry analysis

Human dental pulp stem cells infected with shRNA‐Stathmin lentiviruses, shRNA‐Stathmin lentiviruses + purmorphamine and shRNA‐Ctrl lentiviruses were collected, and after centrifugation, the cells were suspended in 5 mL of cold 70% and incubated at 4°C overnight to fix the cells. The cells were rinsed with PBS, removed from the supernatant after centrifugation and treated with 100 μL of RNaseA (Beyotime, Guangzhou, China) at 37°C for 30 minutes and stained with propidium iodide (Beyotime, Guangzhou, China) in the dark at 4°C for 30 minutes. Then, the cell cycle phases G0/G1, S and G2/M were analysed using a fluorescence activated flow cytometer cell analyser (BD Falcon, San Jose, USA). Three separate experiments were performed.

### 2.7 Real‐time PCR

Total RNA was isolated using Trizol (Takara, Dalian, China), and first‐strand cDNA synthesis was performed according to the PrimeScript RT reagent Kit (Takara). Real‐time PCR was performed with SYBR Premix DimerEraser™ (Takara) using a LightCycler 480 (Roche, Indianapolis, IN, USA). The experiments were repeated in triplicate using human glyceraldehyde‐3‐phosphate dehydrogenase (hGAPDH) as a housekeeping gene to normalize the amount of cDNA. Primer sequences were designed by Invitrogen. Amplification conditions were denaturing at 95°C for 10 minutes, followed by 40 cycles at 95°C for 10 seconds and at 60°C for 30 seconds. The primer sequences used in the experiments are listed in Table 1.

**Table 1:** Primers used for real‐time PCR

Target gene	Primer sequence (5′‐3′)
Stathmin	Forward: TACACTGCCTGTCGCTTGTC
	Reverse: AGGGCTGAGAATCAGCTCAA
Shh	Forward: GCTCGGTGAAAGCAGAGAAC
	Reverse: CCAGGAAAGTGAGGAAGTCG
PTH1	Forward: GCCACAGCCCCTAACAAAAAT
	Reverse: ACCCACAATCAACTCCTCCTGT
SMO	Forward: CTGGTGTGGTTTGGTTTGTG
	Reverse: GGTGAGCAGGTGGAAGTAGG
GLI1	Forward: CTGGACCTGCAGACGGTTATC
	Reverse: AGCCTCCTGGAGATGTGCAT
BSP	Forward: GCGAAGCAGAAGTGGATGAAA
	Reverse: TGCCTCTGTGCTGTTGGTACTG
OCN	Forward: CTCACACTCCTCGCCCTATT
	Reverse: CGCCTGGGTCTCTTCACTAC
ALP	Forward: CCAAAGGCTTCTTCTTGCTG
	Reverse: CCACCAAATGTGAAGACGTG
DSPP	Forward: TTAAATGCCAGTGGAACCAT
	Reverse: ATTCCCTTCTCCCTTGTGAC
GAPDH	Forward: TTCGACAGTCAGCCGCATCTT
	Reverse: ATCCGTTGACTCCGACCTTCA

### 2.8 Western blot

Human dental pulp stem cells transfected with shRNA‐Stathmin lentiviruses, shRNA‐Stathmin lentiviruses + purmorphamine and shRNA‐Ctrl lentiviruses were collected and lysed in SDS‐RIPA buffer containing phenylmethanesulfonyl fluoride (Beyotime, Guangzhou, China). The cell extracts were separated by SDS‐polyacrylamide gel electrophoresis and then transferred onto polyvinylidene difluoride (PVDF) membranes (PVDF, Pall, USA) at 300 mA for 2‐3 hours at 300 mA. The membranes were blocked with 5% skimmed milk in TBS‐T (0.05% Tween 20) and incubated with rabbit anti‐human Stathmin (1:20 000; Abcam), Shh (1:1000; Abcam) or GLI1 (1:1000; Abcam) antibodies overnight at 4°C. After they were washed, the membranes were visualized with horseradish peroxidase‐linked goat anti‐rabbit IgG (1:20 000; Sigma) performed with ECL reagents (Beyotime Biotech, Shanghai, China).

### 2.9 Alizarin red S staining

Human dental pulp stem cells infected with shRNA‐Stathmin lentiviruses and shRNA‐Ctrl lentiviruses were cultured in 6‐well plates with 2 mL of complete culture medium. When the cell density reached 70%, complete medium supplemented with 0.1 μmol/L dexamethasone, 50 μmol/L ascorbic acid and 10 mmol/L β‐glycerol phosphate was used to induce the osteogenic differentiation of the cells for 21 days. After cells were fixed in 4% paraformaldehyde for 10 minutes at room temperature, the induced cells were stained with 0.1% alizarin red S. The assay was performed 3 times.

### 2.10 Statistical analysis

Outcome measurements were analysed and displayed as the mean ± standard deviation. Statistical significance was performed by the two‐sample *t* test using SPSS v.20.0 software (SPSS Inc., Chicago, USA). The Value of *P *<* *.05 was considered as statistically significant.

## 3 RESULTS

### 3.1 Expression of Stathmin in cytomembrane and cytoplasm of hDPSCs

**Figure 1:**
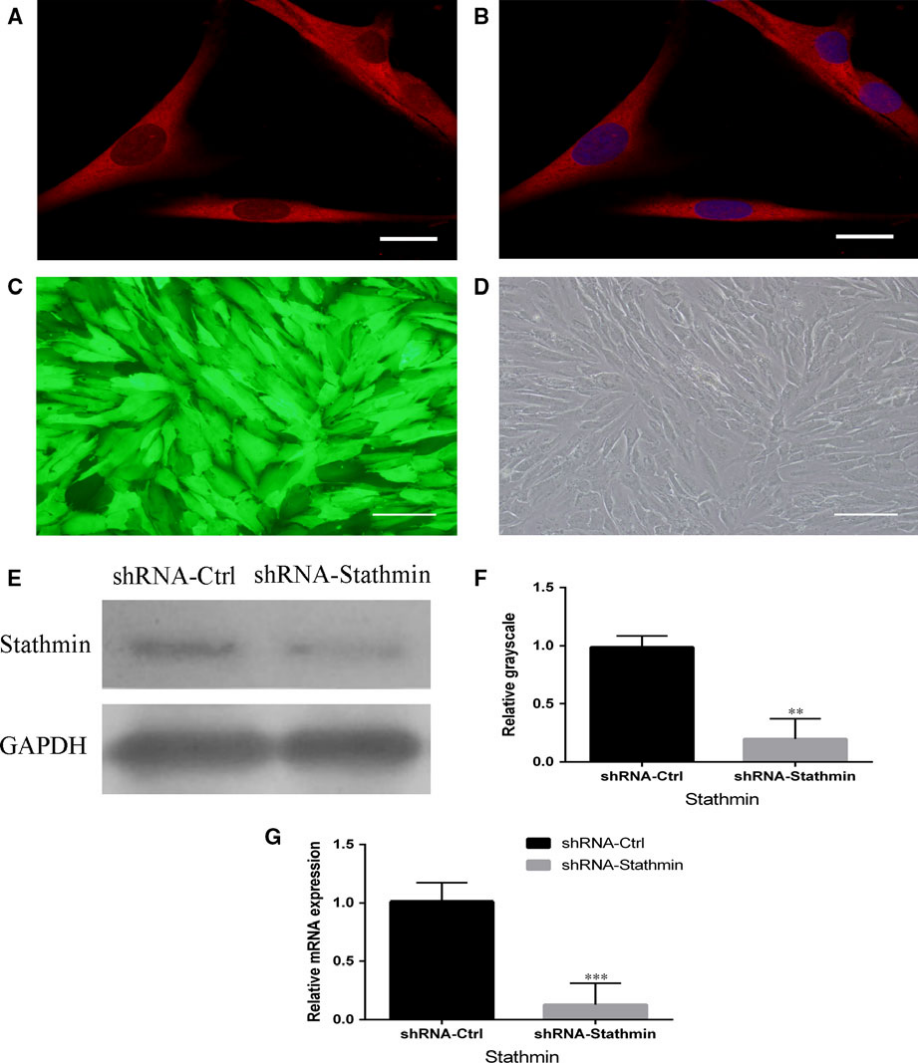
Expression of Stathmin in hDPSCs and lentivirus infections of hDPSCs. A, B, Expression of Stathmin in the cytomembrane and cytoplasm of hDPSCs (scale bar = 20 μm). C, D, At 72 h after gene transduction, both shRNA‐Stathmin‐ and shRNA‐Ctrl‐transfected hDPSCs grew well, and green fluorescent protein (GFP) fluorescence in the hDPSCs confirmed the transfection efficiency (scale bar = 100 μm). E, F, Western blot analysis showing that the Stathmin protein band in the shRNA‐Stathmin group was significantly weaker than that in the shRNA‐Ctrl group. G, Real‐time PCR showed that the expression levels of Stathmin messenger RNA were lower in the shRNA‐Ctrl group than in the shRNA‐Stathmin group (***P <* *.001). Each experiment was repeated in triplicate

Stathmin localization in hDPSCs determined by cell immunofluorescence with the use of confocal microscopy showed that Stathmin was expressed in the cytomembrane and cytoplasm of hDPSCs (Figure 1A,B).

### 3.2 ShRNA lentiviruses inhibit Stathmin messenger RNA and protein expression

Human dental pulp stem cells grew well and were successfully infected with a lentiviral vector encoding Stathmin and GFP. Both the shRNA‐Stathmin group and the shRNA‐Ctrl group showed strongly green fluorescence at 72 hours, with a transfer efficiency of more than 80%. The expression levels of Stathmin mRNA and protein were lower in the Stathmin knockdown cells than in the controls (Figure 1C‐G, **P <* *.01, ***P <* *.001), which showed effective silencing of Stathmin with specific shRNA.

### 3.3 Suppressing of Stathmin in hDPSCs inhibits proliferation

**Figure 2:**
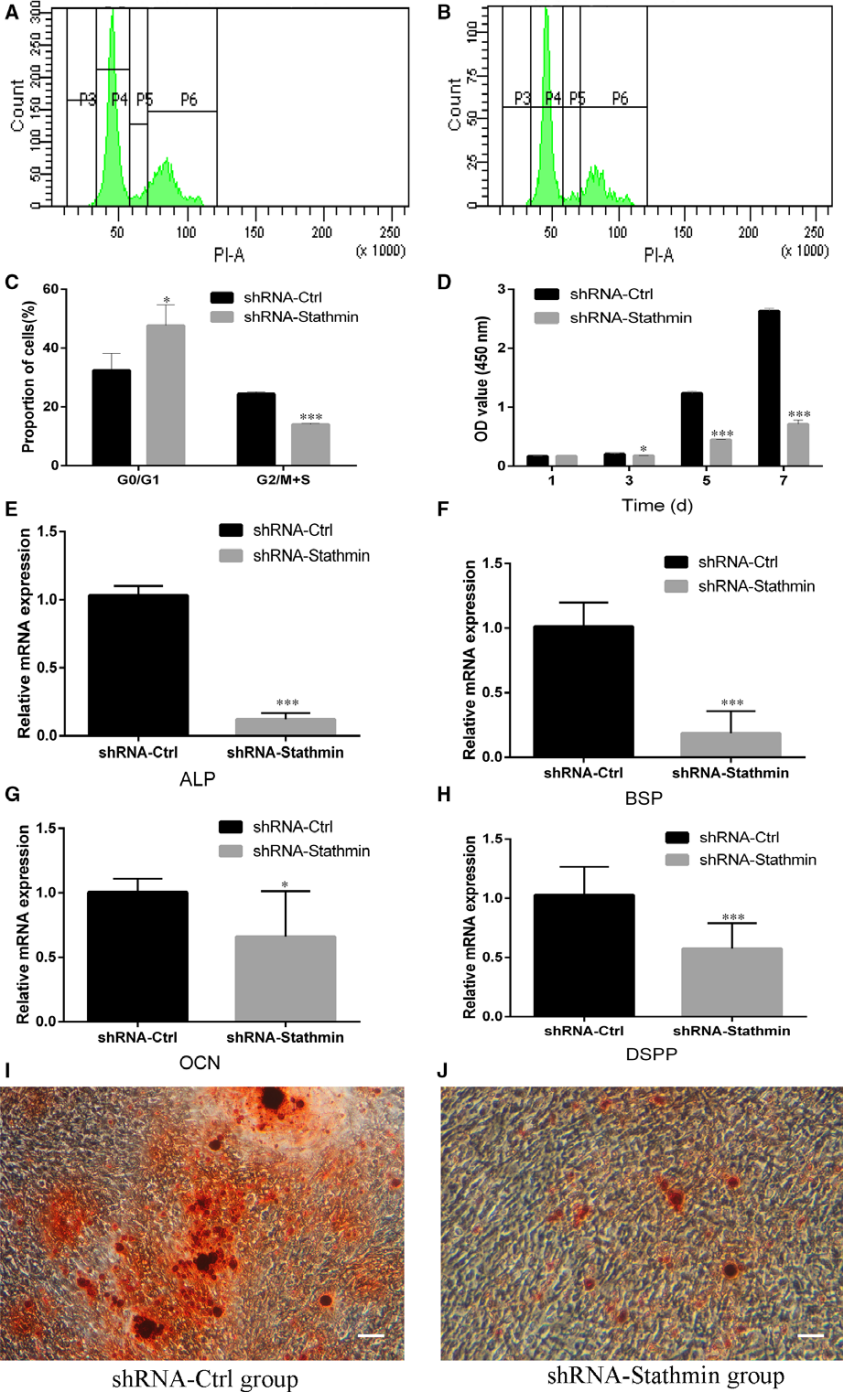
Suppression of Stathmin in hDPSCs inhibited proliferation and the expression of mineralization‐related genes in hDPSCs. A‐C, Cell cycle distribution for the shRNA‐Ctrl group (A) and the shRNA‐Stathmin group (B) and statistical analysis (C). D, CCK‐8 value of shRNA‐Stathmin hDPSCs and shRNA‐Ctrl hDPSCs at days 1, 3, 5 and 7. Mineralization‐related genes (E, ALP; F, BSP; G, OCN; H, DSPP) were expressed at much lower levels in the Stathmin knockdown group than in the shRNA‐Ctrl group cultured in mineralization medium for 3 weeks. I and J, Alizarin red S staining showed that Stathmin suppression significantly inhibited mineral formation by hDPSCs (scale bar = 100 μm). Each experiment was repeated in triplicate. (*P <* *.05, **P <* *.01, ***P <* *.001)

Cell cycle analysis showed that a markedly higher percentage (*P <* *.05) of Stathmin knockdown hDPSCs were in the G0/G1 phase (47.73% ± 7.07%) compared to that in the shRNA‐Ctrl group (32.40% ± 5.81%) (Figure 2A‐C). A CCK‐8 assay performed on cells infected with Stathmin‐specific shRNA lentiviruses at different time‐points (days 1, 3, 5 and 7) after infection demonstrated decreased cell proliferation in the shRNA‐Stathmin group compared with that in the shRNA‐Ctrl group 3, 5 and 7 days after infection (Figure 2D, *P <* *.05, **P <* *.01, ***P <* *.001).

### 3.4 Silencing of Stathmin in hDPSCs blunts osteo‐/odontogenic differentiation

To explore the role of Stathmin in hDPSC osteo/odontogenic differentiation, hDPSCs in both the shRNA‐Stathmin group and the shRNA‐Ctrl group were cultured in mineralization medium for 3 weeks. The mRNA expression levels of ALP, BSP, OCN and DSPP were detected by real‐time RT‐PCR assays. Lower expression levels of the osteogenic/odontoblastic differentiation markers were seen in the shRNA‐Stathmin group compared to that in the control group (Figure 2E‐H, *P <* *.05, ***P <* *.001).

Alizarin red S staining showed obvious bone‐like nodules with lower numbers of calcium nodules in shRNA‐Stathmin hDPSCs than in the shRNA‐Ctrl group (Figure 2I,J).

### 3.5 Silencing of Stathmin inhibits Shh signalling pathway

**Figure 3:**
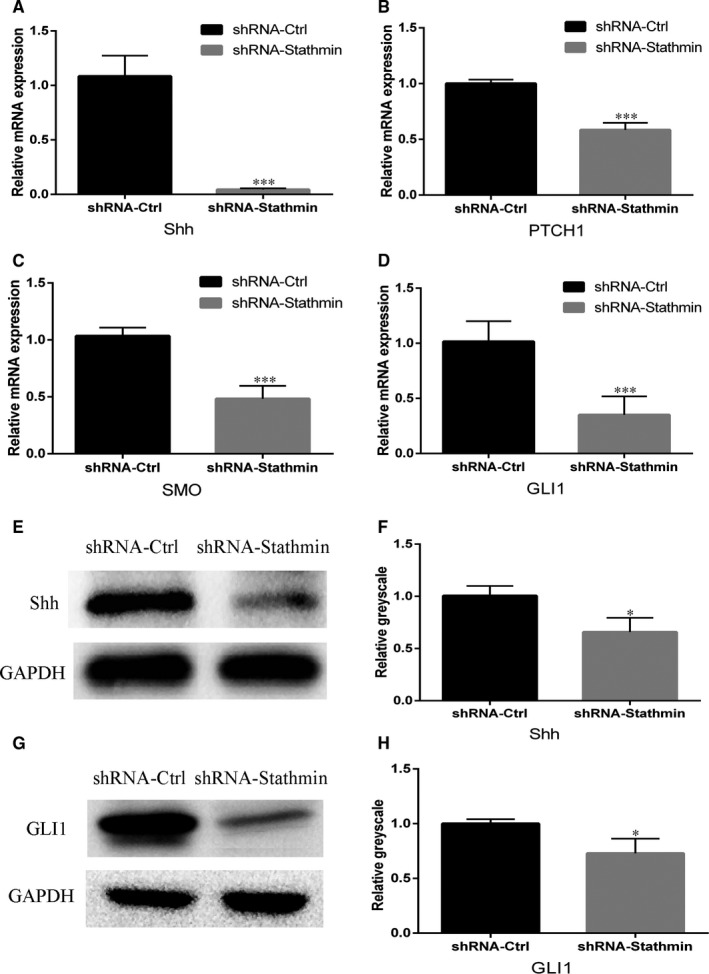
Silencing of Stathmin inhibits the Shh signalling pathway. A‐D, Real‐time PCR analysis of the Shh signalling pathway (A, Shh; B, PTCH1; C, SMO; D, GLI1; ***P <* *.001). E‐H, Western blot analysis showing that the expression levels of the Shh and GLI1 proteins were much lower in the shRNA‐Stathmin hDPSCs than in the shRNA‐Ctrl hDPSCs. Each experiment was repeated in triplicate

Real‐time RT‐PCR assays used to test the expression of Shh and its downstream transcription factor showed that the expression of Shh was significantly decreased in the shRNA‐Stathmin group compared to that the shRNA‐Ctrl group (Figure 3A, ***P <* *.001). The expression of PTCH‐1, SMO and GLI1 was also decreased after shRNA‐Stathmin transduction (Figure 3B‐D, ***P <* *.001). Western blot results confirmed that Shh and GLI1 were decreased after the silencing of Stathmin (Figure 3E‐H).

### 3.6 Activation of the Shh promotes osteogenic/odontoblastic differentiation of hDPSCs

**Figure 4:**
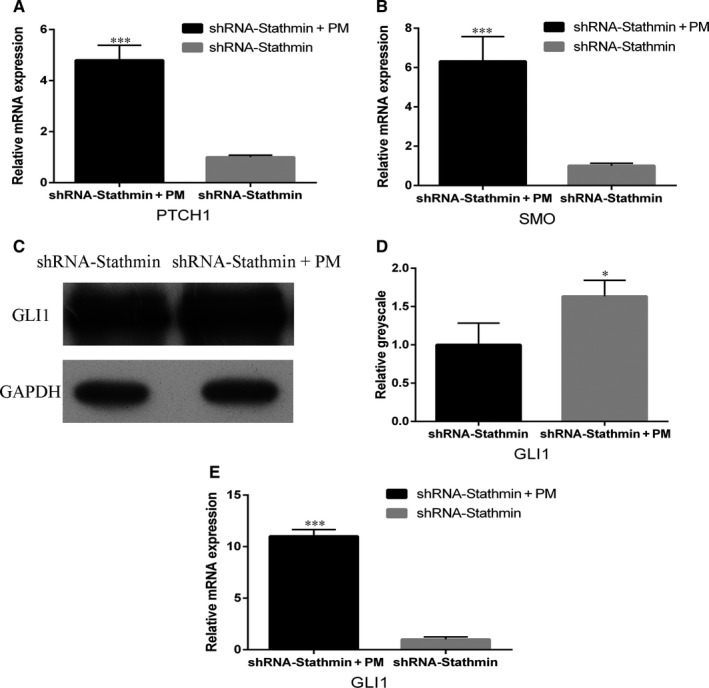
Purmorphamine treatment analysis. Real‐time PCR showed that the Shh signalling pathway was activated in the presence of purmorphamine and (A, PTCH1; B, SMO; E, GLI1; ***P <* *.001) expression was up‐regulated. Protein expression (C, D) supported the activation of GLI1. Each experiment was repeated in triplicate

**Figure 5:**
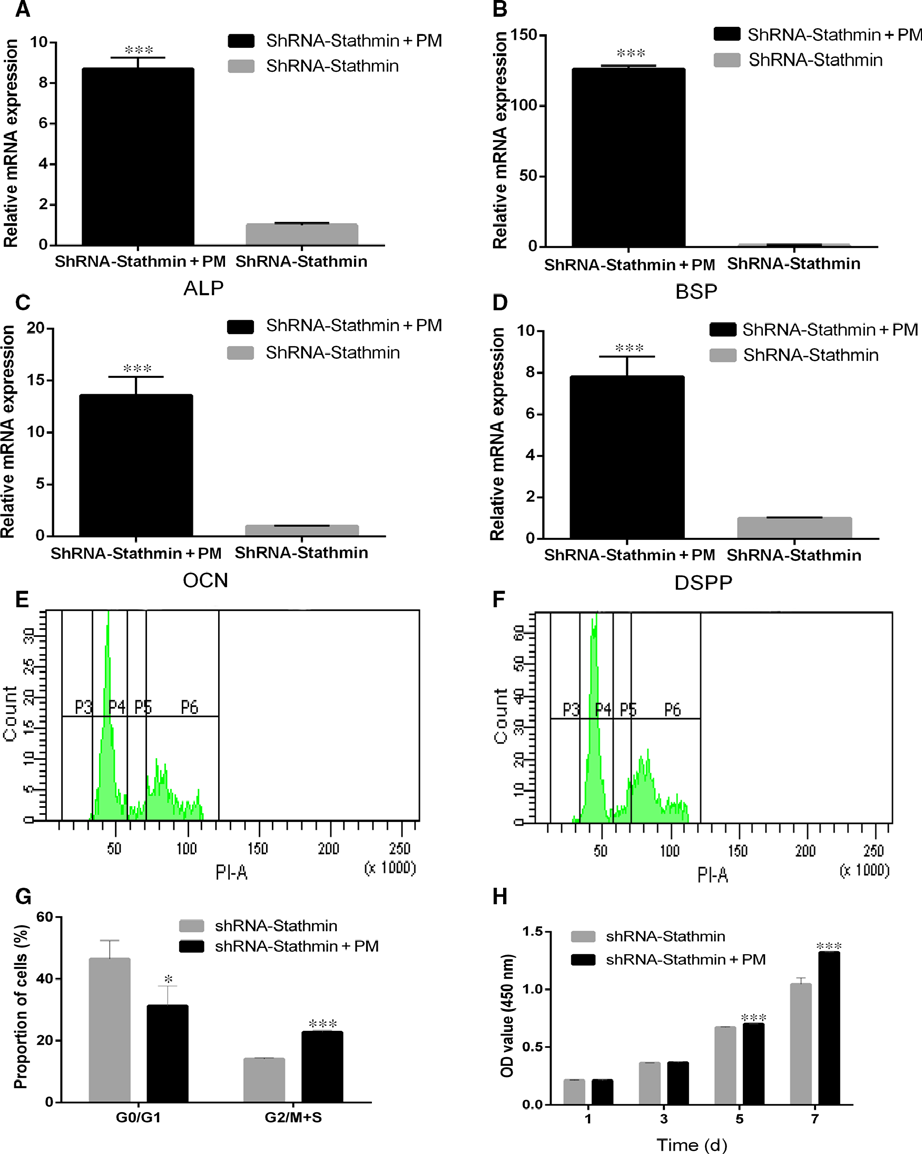
Activation of Shh signalling promotes the proliferation and osteogenic/odontoblastic differentiation of hDPSCs. A‐D, Treatment of shRNA‐Stathmin hDPSCs with purmorphamine that specifically binds SMO increased (A, ALP; B, BSP; C, OCN; D, DSPP) mRNA expression, as determined by real‐time PCR. E‐G, Cell cycle distribution for the shRNA‐Stathmin group (E) and the shRNA‐Stathmin + PM group (F) and statistical analysis (G). H, CCK‐8 values of shRNA‐Stathmin + PM hDPSCs and shRNA‐Stathmin hDPSCs at days 1, 3, 5 and 7. Each experiment was repeated in triplicate (*P <* *.05, ***P <* *.001)

Purmorphamine activation is known to induce Shh^25^; therefore, hDPSCs were treated with purmorphamine after Stathmin was silenced. The real‐time PCR and Western blotting results showed that PTCH1, SMO and GLI1 expression levels were up‐regulated compared with those in the shRNA‐Stathmin control group (Figure 4A‐E, ***P <* *.001), confirming that purmorphamine activated the Shh signalling pathway in hDPSCs after transduction with Stathmin shRNA lentiviruses. Activation of Shh signalling also increased ALP, BSP, OCN and DSPP expression (Figure 5A‐D, ***P <* *.001), supporting a role for Shh signalling in osteogenic/odontoblastic differentiation of hDPSCs.

### 3.7 Activation of Shh signalling pathway promotes proliferation of hDPSCs

We then determined whether activation of the Shh signalling pathway is sufficient to promote hDPSC proliferation. To achieve this goal, purmorphamine was added to hDPSCs transfected with silencing Stathmin expression lentiviruses, and cell cycle and CCK8 assays analyses were used to detect the proliferation of hDPSCs. The results of the cell cycle analysis also proved that a lower percentage (*P <* *.05) of hDPSCs in the shRNA‐Stathmin + PM group were in the G0/G1 phase (31.27%% ± 6.45%) compared with hDPSCs in the shRNA‐Stathmin group (46.40% ± 6.05%) (Figure 5E‐G). The CCK8 results showed that the proliferation rate of hDPSCs in the shRNA‐Stathmin + PM group was significantly higher than that of the shRNA‐Stathmin group on days 5 and 7 (Figure 5H, ***P <* *.001).

## 4 DISCUSSION

The identification of mechanisms of adult DPSC osteogenic/odontogenic differentiation is of prime interest for developing therapeutic strategies for tooth formation and regeneration. In this study, we established the essential role of Stathmin in hDPSC osteogenic/odontogenic differentiation and indicated that specific activation of this molecule in hDPSCs promotes osteogenic/odontogenic differentiation. This finding supplies an effective method to promote hDPSC osteogenic/odontogenic capacity, which may be exploited for tooth regeneration.

The Stathmin phosphoprotein family consists of stathmin, SCLIP, SCG10, RB3 and two variants of RB3, RB3′ and RB3″. Members of this family are widely expressed in various types of cells. Although the expression level of Stathmin is different in different cell types, it has been found that the expression of the Stathmin protein in well‐differentiated cells is significantly reduced.^26^ Immunocytochemistry results show that in poorly differentiated hDPSCs, Stathmin is distributed in the cytoplasm and the cell membrane and maintains a strong fluorescence signal.

We first identified the effect of Stathmin on osteogenic/odontogenic differentiation in hDPSCs by constructing a lentiviral vector for Stathmin silencing analysis. We showed that reduced expression of Stathmin was sufficient to block osteogenic/odontogenic differentiation in hDPSCs. Notably, reduced expression of Stathmin decreased the expression of ALP, which plays a role in bone matrix mineralization and has been used as an early marker gene during osteogenesis and dentinogenesis;^27^, ^28^ BSP, a significant component of the bone extracellular matrix; OCN, a glycoprotein that binds calcium and is secreted by osteoblasts during bone formation, initiating mineralization and promoting mineral crystals;^29^ and DSPP, one of the most important markers of odontoblasts. Consistent with this finding, Stathmin‐specific silencing reduced the mineralization identified by Alizarin red S staining. Taken together, these data indicate that Stathmin is important for the osteogenic/odontogenic differentiation of hDPSCs in vitro.

Hedgehog (Hh) is a secreted signal molecule capable of regulating all stages of embryo development and the formation of a variety of tissues and organs.^30^ Shh is a member of the vertebrate Hedgehog family, and some studies have shown that Shh plays a role in whole tooth formation.^31^ However, the role of the Shh signalling pathway in the regulation of osteogenic/odontogenic differentiation of hDPSCs by Stathmin has not been reported. To examine whether the Shh signalling pathway is involved in Stathmin regulation, we investigated the changes of Shh and its two transmembrane receptors PTCH1 and SMO, as well as changes of the nuclear transcription factor GLI1, after silencing Stathmin. The nuclear transcription factor GLI1 is not only associated with the target gene of the effector but also serves as a positive feedback reporter of the Shh ligand protein and is widely recognized as an important and reliable marker for monitoring the activity of the Shh signalling pathway.^32^, ^33^ In this study, it was found by real‐time PCR and Western blotting that the expression of Shh, PTCH1, SMO and GLI1 was significantly decreased after Stathmin expression was inhibited, suggesting that the Shh/GLI1 signalling pathway plays a role in the regulation of hDPSCs osteogenic/odontogenic differentiation by Stathmin.

The CCK8 assay and cell cycle analysis showed that the proliferation of hDPSCs in the shRNA‐Stathmin group was significantly decreased compared to that in the shRNA‐Ctrl group. Stathmin is the substrate of a variety of intracellular kinases, which can regulate the cell cycle by regulating the dynamic balance of the microtubule system. The downstream target of Stathmin is a type of tubulin, microtubule and spindle organization, which plays a key role in the cell division and thus changes the biological behaviour of cell proliferation and differentiation. At present, the effect of Stathmin on cell proliferation has mainly focused on its role in tumour cell lines, with few studies carried out in stem cells. It was found that the expression of Stathmin in acute leukaemia cells was significantly increased, while the proliferation, survival and clonal ability of cancer cells were significantly decreased in breast cancer cell lines with inhibited Stathmin expression.^34^, ^35^ The expression of Shh and its downstream signalling molecules were significantly decreased after Stathmin inhibition. It was reported that Shh could promote the proliferation of bone marrow mesenchymal stem cells, dental pulp cells and periodontal ligament cells.^36^, ^37^ Collectively, both Stathmin and Shh could decrease the proliferation of DPSCs after Stathmin expression was silenced, which is consistent with our results, suggesting that Stathmin may simultaneously control the proliferation of hDPSCs by participating in the Shh signalling pathway.

Purmorphamine is a chemically synthesized purine derivative. It has been reported in mouse cell line studies that purmorphamine activates the downstream transcription factor GLI1 of the Hh signalling pathway by directly acting on the membrane receptor Smoothened (SMO).^38^ To further investigate the role of the Shh signalling pathway in Stathmin's involvement in the regulation of proliferation and osteogenic/odontoblastic differentiation of hDPSCs, we added the Shh signalling pathway activator purmorphamine to hDPSCs transfected with Stathmin silencing lentiviruses. In this study, the purmorphamine activation concentration was based on the research results of Wu,^39^ who used the mouse mesenchymal cell line C3H10T1/2 to determine that the EC50 of purmorphamine was 1 μmol/L when the activity of ALP was used as a reference standard. Real‐time PCR and Western blotting showed that the expression levels of SMO, PTCH1 and GLI1 were significantly increased after purmorphamine was added to hDPSCs with silenced Stathmin expression, indicating that the activation of the Shh signalling pathway was effective. Real‐time PCR results showed that compared to hDPSCs in the shRNA‐Stathmin group, the expression of ALP, BSP, OCN and DSPP was significantly up‐regulated in the shRNA‐Stathmin + PM group, indicating that the activation of the Shh signalling pathway can re‐induce the osteogenic/odontogenic differentiation of hDPSCs.

CCK8 results showed that the proliferation of hDPSCs in the shRNA‐Stathmin + PM group was significantly higher than that in the shRNA‐Stathmin group on days 5 and 7. The reason for this result may be that purmorphamine requires a certain period of time to counteract the Stathmin‐induced inhibition of hDPSC proliferation. Cell cycle analysis data demonstrated the proliferation of hDPSCs was markedly increased and that fewer cells remained in the G0/G1 cell cycle phase in the presence of purmorphamine. In the present study, the effect of purmorphamine on human cell proliferation remains controversial. This may be related to the difference in the effect of purmorphamine on cell types and cell culture medium of different origins. In Beloti's study,^40^ it was found that purmorphamine did not affect the proliferation of human bone marrow mesenchymal cells and osteoblast differentiation but that it increased ALP expression and the ability of cells to produce calcium nodules. However, Rezia and others^41^ found that the addition of purmorphamine could promote the proliferation of hDPSCs, consistent with the results of this experiment.

Although this study confirmed that the inhibition of Stathmin expression could inhibit the proliferation and osteogenic/odontoblastic differentiation of hDPSCs through the Shh signalling pathway, the method of Stathmin overexpression needs to be further validated. Additionally, after clarification of the effect of Stathmin on hDPSCs cultured in vitro, in vivo experiments will be performed.

In summary, this study demonstrated that Stathmin plays a positive role in hDPSC proliferation and osteogenic/odontoblastic differentiation and that its regulation may be mediated by the Shh/GLI1 signalling pathway. This study provides a theoretical basis for further study of the dentin differentiation mechanism of DPSCs and provides avenues, for example if Stathmin as a key gene can apply in clinic to repair pulp tissue,for further research needed to determine if there are other signalling pathways involved.

## CONFLICT OF INTERESTS

The authors confirm that there are no conflict of interests.

## AUTHOR CONTRIBUTIONS

Haiyue Yu Performed the research; Dandan Ma and Buling Wu designed the research study; He Wang and Xiaoyi Zhang contributed essential reagents or tools; Shuaimei Xu and Tingting Ning analysed the data; Dandan Ma and Haiyue Yu wrote the manuscript.
